# Personal Electronic Health Records: Understanding User Requirements and Needs in Chronic Cancer Care

**DOI:** 10.2196/jmir.3884

**Published:** 2015-05-21

**Authors:** Ines Baudendistel, Eva Winkler, Martina Kamradt, Gerda Längst, Felicitas Eckrich, Oliver Heinze, Bjoern Bergh, Joachim Szecsenyi, Dominik Ose

**Affiliations:** ^1^University Hospital HeidelbergDepartment of General Practice and Health Services ResearchHeidelbergGermany; ^2^National Center for Tumor Diseases (NCT)Ethics and Patient-Oriented CareHeidelbergGermany; ^3^University Hospital HeidelbergDepartment of Information Technology and Medical EngineeringHeidelbergGermany

**Keywords:** personal electronic health record, user requirements, functions, colorectal cancer, chronic care

## Abstract

**Background:**

The integration of new information and communication technologies (ICTs) is becoming increasingly important in reorganizing health care. Adapting ICTs as supportive tools to users' needs and daily practices is vital for adoption and use.

**Objective:**

In order to develop a Web-based personal electronic health record (PEPA), we explored user requirements and needs with regard to desired information and functions.

**Methods:**

A qualitative study across health care sectors and health professions was conducted in a regional health care setting in Germany. Overall, 10 semistructured focus groups were performed, collecting views of 3 prospective user groups: patients with colorectal cancer (n=12) and representatives from patient support groups (n=2), physicians (n=17), and non-medical HCPs (n=16). Data were audio- and videotaped, transcribed verbatim, and thematically analyzed using qualitative content analysis.

**Results:**

For both patients and HCPs, it was central to have a tool representing the chronology of illness and its care processes, for example, patients wanted to track their long-term laboratory findings (eg, tumor markers). Designing health information in a patient accessible way was highlighted as important. Users wanted to have general and tumor-specific health information available in a PEPA. Functions such as filtering information and adding information by patients (eg, on their well-being or electronic communication with HCPs via email) were discussed.

**Conclusions:**

In order to develop a patient/user centered tool that is tailored to user needs, it is essential to address their perspectives. A challenge for implementation will be how to design PEPA’s health data in a patient accessible way. Adequate patient support and technical advice for users have to be addressed.

## Introduction

The integration of new information and communication technologies (ICTs) is becoming increasingly important in reshaping the way health care is understood and delivered [1]. Significant potential is seen in ICT concepts aiming to give patients access to their own health- and treatment-related information [2-5]. In particular, personal health records (PHRs) are seen as promising tools, ranging from standalone to tethered to integrated approaches [6,7]. PHR systems that often used in the United States allow patients to access primary data from an electronic health record managed by health care professionals (HCPs) through a patient portal (tethered PHRs) [8].

However, design and implementation of innovative patient-centered PHRs has not proven to be easy. Experiences from nationwide approaches such as the National Health Service implemented personal electronic health record HealthSpace (England) show that it failed due to a lack of usefulness and user friendliness, as well as poor alignment to users’ expectations and self-management practices [9]. According to adoption and use, user orientation in ICT development, implementation, and evaluation is central [2,9].

Moreover, current literature shows that patients and health care professionals may have complementary perspectives regarding PHRs. In general, patients do have a positive attitude towards the use of a PHR [10-13] and are willing to share their health-related information via new ICTs [10,14-16]. However, health care professionals more often express concerns regarding PHRs instead of discussing possible benefits [13,17-19].

In our research project, we are developing a patient-controlled personal electronic health record (PEPA) in chronic care of patients with colorectal cancer. As a subset of PHRs, the Web-based PEPA would enable patients to access, maintain, and manage (including access management) a secure copy of their personal health information integrated from various HCP primary systems (eg, electronic medical records in hospital, electronic health records in general practice). Within the PEPA concept, patients are understood to be active partners who manage their personal health information across health care settings.

For an innovative ICT like PEPA to create added benefit and function as a supportive tool in managing complex chronic illness and care, it is essential that it fit into the real world, daily practices, and health care structures of patients with cancer and their HCPs. Therefore, it is important to better understand the needs and requirements of prospective users. The aim of this study was to explore needs and requirements of potential users with regard to the content and function of a PEPA.

## Methods

### Study Design

A pilot project called “Information technology for patient-centered health care” (INFOPAT), funded by the German Federal Ministry of Education and Research (2012-16), has been initiated in the Rhine-Neckar region (population: 2.3 million) in Germany aiming to improve cross-sectoral health care especially for patients with colorectal cancer. Within this project, a PEPA is being developed and implemented regionally.

In the first phase of this INFOPAT-project, a qualitative, exploratory study design using focus groups was chosen, to allow intensive exploration of requirements and needs of selected user groups. The following general research questions were explored within this analysis: (1) What requirements do potential users have regarding the PEPA content?, (2) What information do potential users perceive as relevant to have available in the PEPA?, and (3) Which PEPA functions do potential users perceive as useful?

Ethical approval was given by the Ethics Committee of the University Hospital Heidelberg (S-497-2012). All participants gave their written informed consent. The participants’ anonymity and confidentiality was ensured throughout the study.

### Study Sample

In a regional (Rhine-Neckar region in Baden-Wuerttemberg, Germany), cross-sectoral health care setting, prospective user groups of a PEPA were identified. The first user group comprised patients with colorectal cancer (ECOG Performance Status 0-1 [20]) as well as representatives (staff) from patient support groups. The second group was made up of physicians and the third group comprised other non-medical HCPs. Patients who fulfilled the following criteria were excluded: younger than 18 years, suffering from severe acute psychiatric disorders as well as moderately to severe dementia.

Patients were recruited through the National Center for Tumor Diseases (NCT) in Heidelberg, Germany, where they received their cancer treatment. Additionally, patients were recruited via an umbrella organization, Heidelberger Selbsthilfebüro, for patient support groups in Heidelberg. Clinicians (oncological specialists) and other non-medical HCPs (nurses, stoma therapist, social services, physiotherapists, and nutritionists) were also recruited at the NCT. General practitioners (GPs), registered medical specialists (eg, oncologists), and health care assistants from GP practices were recruited by the Department of General Practice and Health Services Research (University Hospital Heidelberg).

### Data Collection

The decision to collect data through focus groups was based on the explorative character of the research topic. A focus group is a kind of group interview with participants who are involved in the research field of interest. The group processes that are evoked by focus groups can help participants explore and clarify their views, attitudes, and opinions, which would be less accessible in a one-to-one interview [21].

From March until October 2013, 10 focus groups with a total of 47 participants were conducted. For all user groups, separate focus groups were performed (3 with patients; 4 with physicians; 3 with other HCPs) (Table 1). On average, the focus groups lasted 120 minutes and took place in rooms at the University Hospital Heidelberg. All data were audio- and videotaped and transcribed verbatim.

An experienced researcher used a semistructured, pilot-tested interview guide based on a literature review and expert discussions for conducting the focus groups. At the beginning of the focus group, a small amount of information was given on the PEPA concept to all participants. At all focus groups, the moderator was supported by a co-moderator. A third researcher wrote a protocol that was integrated into the data analysis phase of this study. The focus group discussions lasted until the saturation of theoretical arguments was reached.

**Table 1:** Composition of focus groups (N=10).

User group	Focus groups, n	Participants (total), n	Description
Patients	3	14	Patients with colorectal cancer, representatives from patient support groups
Physicians	4	17	Oncological specialists, GPs, registered specialists
Other HCPs	3	16	Nurses, health care assistants, social services, nutritionists, physiotherapists
Total	10	47	

### Data Analysis

The approach for the descriptive qualitative analysis used in this study [22,23] dealt with the transcribed texts of conducted focus groups as material, in which all data were embedded. To perform a qualitative content analysis, data were taken from the transcripts, edited, and analyzed. This was done by using a preliminary category system as search grid. The preliminary category system was based on theoretical considerations, expert discussions, and a literature review. During the entire process of analysis, the category system was adapted if the data revealed additional and new information that did not fit into the previous category system.

Therefore, the performed qualitative content analysis included inductive development of categories and a deductive application of categories. In a first step, three transcripts were reviewed independently by the first author (IB), a coauthor (MK), and the last author (DO) using the preliminary category system and additional key issues were identified. After summarizing and labeling key issues as codes, the codes were sorted into main and subcategories [23]. The codes were clearly defined and linked with representative examples from the original texts. The categories were discussed and further modified within the interprofessional researcher team until a consensus on the category system was achieved. Qualitative content analysis of the data was performed using the software ATLAS.ti (version 7.0.80).

### Presentation of Results

In order to facilitate better readability, the key findings are presented in categories, subcategories, and aspects. Tables that present the categories enable differentiation between the user groups’ perspectives with respective aspects mentioned. The quotations cited in this article were cross-checked by a native speaker in the Department of General Practice and Health Services Research after translation from German into English.

## Results

### Overview

Table 2 summarizes the characteristics of patients with colorectal cancer (n=12), representatives from patient support groups (n=2), physicians (GPs, registered specialists, oncological specialist) (n=17), and 16 other non-medical HCPs like nurses including a stoma therapist (n=7), health care assistants (n=4), social services (n=2), nutritionist (n=1), and physiotherapists (n=2).

**Figure 1:**
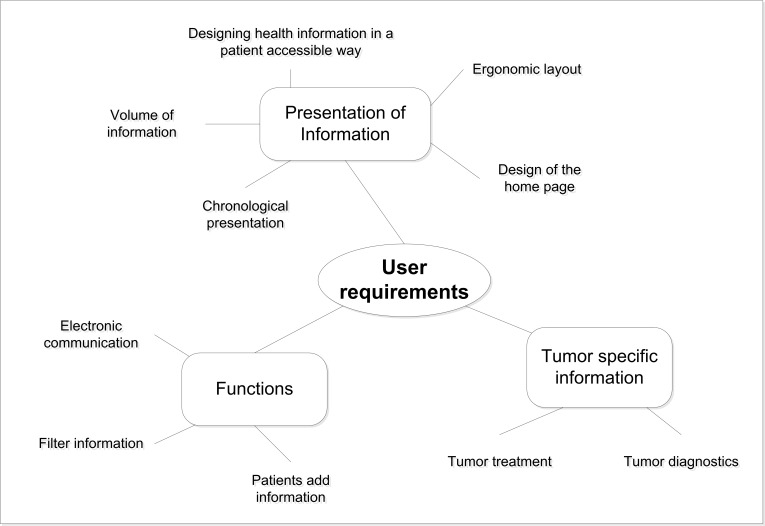
Overview of identified key results regarding user requirements.

Overall, the key results presented here show that focus group participants discussed user requirements like the presentation of information, tumor specific information that is needed, and possible useful functions (Figure 1).

**Table 2:** Sample characteristics of focus group participants (N=47).

		Patients (n=12)	HCPs (n=16)	Physicians (n=17)	Patient representatives^a^(n=2)
Sex (male), % (n)		83.3 (10)	18.8 (3)	58.8 (10)	50.0 (1)
Age (years), median (interquartile range)		61.5 (58.0-67.2)	38.0 (28.5-50.0)	43.0 (35-56.5)	(44;62)^b^
Living in rural area^c^, % (n)		58.3 (7)	—	—	—
Living with a partner, % (n)		91.7 (11)	—	—	—
Education ≥12 years, % (n)		50.0 (6)	43.7 (7)	—	100.0 (2)
Duration since diagnosis (years), median (interquartile range)		1.7 (0.8-6.7)	—	—	—
Professional experience (years), median (interquartile range)		—	20 (5.0-26.0)	15 (5.0-26.5)	(10;38)^b^
**Living with diagnosis, % (n)**					
	<1 year	33.3 (4)	—	—	—
	1-2 years	33.3 (4)	—	—	—
	≥6 years	33.3 (4)	—	—	—
**Health care setting, % (n)**					
	NCT	—	75 (12)	29.4 (5)	—
	Outpatient care^d^	—	25 (4)	70.6 (12)	—

^a^Patient representatives=staff from patient support groups.

^b^Minimum; maximum.

^c^Less than 15,000 inhabitants.

^d^General practitioners; registered specialists.

### User Requirements: Personal Electronic Health Record Information and its Presentation

#### Overview

During the focus group discussions, issues on PEPA information and how it should be presented in a PEPA were central in all groups. The following user requirements on these issues were identified (Table 3).

**Table 3:** User requirements: PEPA information and its presentation.

Subcategory	Aspects	User group^a^
Volume of PEPA information	Need for complete data	b
	Manageability of large amounts of data	a/b
	Need for time and content-related limits	a/b/c
Designing health information in a patient accessible way	Information comprehensible to laypersons	a/b/c
	Adapting the presentation of medical results	b
	Glossary to support comprehensibility	a/c
Chronological presentation of illness related information	Tracking the course of illness and treatment	a/b/c
	Information in chronological order	a/b/c
	Identifying and utilizing unstructured information	b
	Tracking long-term laboratory findings	a
Ergonomic layout	Clarity	b/c
	Ease of use	b/c
Design of the home page	Key information on the home page	a/b/c
	Priority for current issues	a/b
	Highlighting entries	b/c

^a^User group: a=patients; b=physicians; c=other HCPs (eg, nurses).

#### Volume of Information in a Personal Electronic Health Record

The volume of information that should be provided in a PEPA was discussed in all three user groups. Different needs regarding this issue emerged. In particular, participants from the physicians’ focus groups expressed their need for complete data in a PEPA. They wished to have information available in the PEPA as much as possible: “I always expect all information from such a record” (GP1-F10).

On the other hand, other participants within the physicians’ group and patients saw a challenge in manageability of large amounts of data: “It’s just such an enormous thing…can you still manage it, do you want to manage it—everyone will have this huge thing to maintain” (Patient4-F01).

In all three user groups, the need for time and content-related limits in information volume was mentioned. Issues on limiting the volume with a timeline were addressed especially by patients, for example, only data from the last 10 years, although they were unsure about when to draw the line. Some patients mentioned that they did not want to have every detailed laboratory result in their PEPA. Similarly, participants from both professional user groups (medical and other HCPs) thought it would be useful to limit the amount of medical results for practical reasons: “Yes, but not everything…I don’t need all 20 of those little blood exams” (Patient4-F01) and “It would perhaps be quite good if only a certain number of the results were in it” (HCP-F06).

#### Designing Health Information in a Patient Accessible Way

A central issue was how to present information in a PEPA for patients. Designing health information in a patient accessible way was discussed by all three user groups. Several patients emphasized their need for having comprehensible information in their PEPA. They highlighted the fact that patients are mostly laypersons who typically have problems understanding medical jargon. One patient suggested a copy of the record that is reworded in patient assessable language while realizing that the associated effort was significant: “I’ve often wished I had a, what shall I call it—a patient copy…to me that’s something that summarizes the most important events of my own life in terms of illness, translated into a language that I can understand” (Patient2-F03).

Similarly, the HCPs user group saw the need for changing the way of presenting medical information in this patient-owned tool, and participants from the physicians’ focus groups also required adapting the presentation of medical results to the patients’ needs: “How the results are displayed needs to be changed completely so that it can be used for the patients” (Physician 3-F04).

Moreover, options were discussed for supporting the comprehensibility of medical terms in a PEPA for patients by the patient and non-medical HCPs focus groups. The usefulness of a glossary, especially in handling medical abbreviations was stressed:

But with the speed…at which the information is given in a one-off appointment, I at least am not able to take it all in. And then I sit at home and really don’t know what this RT, all these abbreviations, what that means. Such a kind of glossary would be extremely helpful here.

Patient2-F03

#### Chronological Presentation of Illness-Related Information

During the focus group discussions, in all three user groups, themes related to chronological information recurrently cropped up. For patients as well as their HCPs, it was important that a PEPA structure would facilitate tracking the course of illness and treatment over time: “First of all, I want to see my medical history shown completely for myself” (Patient1-F02) and “I also think that it would be important to have the data in a way that you can track the progress/ process quite quickly” (GP1-F09).

Some physicians explicitly highlighted the need for presenting the PEPA information in chronological order for their daily practice in treating patients with colorectal cancer but concurrently stressed the big challenge in technological practicability:

The challenge certainly lies in putting individual results into a useful chronology.

Physician3-F04

I think it’s sensible to categorize it (the information) and assign it to the different areas so that you really end up with this chronology…he had the last chemotherapy on day xy and this was the medication and a week ago he took a break because his blood values were bad.

Physician3-F04

A further issue pointed out in the physician focus groups was that physicians dealt with patients’ health information in electronic patient records within their daily work where, currently a lot of necessary health information is provided in an unstructured way, for example, in medical reports or physician’s letters (as PDF files). From their perspective, a PEPA would have a real benefit for their daily work if it could identify and utilize unstructured information from various information systems and present it in a structured form in the PEPA:

because if it can’t do that, I end up with what feels like ten PDF files from the patient and one is the doctor’s letter from the University Hospital and one is the doctor’s letter from the oncologist and the third PDF is maybe a copy of a prescription…The question is, how do we make the new system more than just the scanned-in doctor’s letters.

Physician3-F04

Patients focused more on the possibility of observing how their condition developed over time (eg, tumor markers). They would benefit from using a PEPA, for example, if tracking their long-term laboratory findings would be possible: “My tumor marker values, for instance, it would be really interesting to see how they progress” (Patient4-F01).

#### Ergonomic Layout

In general, issues with PEPAs related to design and layout were not central to discussions. In particular, patients did not have a detailed idea on how the PEPA layout and its user interface should appear: “The design isn’t that important to me” (Patient4-F01). However, participants from both professional user groups (medical and other HCPs) had several overall requirements regarding this issue. Both wished that a PEPA would have an ergonomic layout that is clearly structured: “The whole thing…really needs to be completely clear” (GP1-F05). Furthermore, the importance of the PEPA’s ease of use for a broad range of users was emphasized: “It needs to be very simple” (GP4-F05) and “understandable to everyone” (HCP1-F07).

#### Design of the Front Page

Regarding the design of the PEPA’s front page, participants from all three user groups, especially physicians mentioned that the information presented at the frontpage should be restricted to key information like administrative data, contact persons, diagnoses, or the current medication plan. Further information of interest they would like to open on sub-levels: “Really the most important...I mean name, address, relatives, telephone number, allergies, diagnoses, maybe the last medication plan—that’s it. And you can open the rest if you want to” (GP2-F05).

Some participants from the patient and the physician user groups pointed out a priority for current issues. They wished to view current information first on the front page. Some participants from both professional user groups (medical and other HCPs) suggested even highlighting important or new information, for example, in terms of color to highlight relevance.

If new entries are added, they need to be specially marked, not integrated into the mails, so that I don’t have to search and read through everything again to find out what’s new. It should be introduced somehow and indicated that it’s new.

Patient6-F03

that some things are highlighted in a different colour, important things.

HCP6-F06

### Relevant Information in a Personal Electronic Health Record From Users’ Perspective

Tumor-specific information was identified as relevant to have available in the PEPA for care delivery in patients with colorectal cancer. Particularly, participants in the physicians focus groups focused on tumor-specific information related to tumor diagnostics and tumor treatment (Table 4).

**Table 4:** User requirements on available tumor specific information in the PEPA.

Categories	Contents	Specifics
Tumor diagnostics	Tumor diagnosis	Initial diagnosis including the date of assessment
	Tumor localization	Tumor localization including the date of assessment
	Tumor laboratory	Tumor marker
	Tumor stage	Information on tumor stage and metastases (TNM-classification); including the date of assessment; and staging or planned staging
Tumor treatment	Chemotherapy	Information on the application of chemotherapy; dose reduction; and status of chemotherapy
	Radiotherapy	Information on dose of radiotherapy

Moreover, general information was identified from the users’ perspective as relevant to have available in the PEPA (see Multimedia Appendix 1). The focus group participants from all three user groups required that a PEPA should provide at least a basic dataset of relevant information that would be crucial for everyone involved in the patients’ health care. Besides administrative data, information like allergies, diagnoses, and medication were highlighted as relevant. Patients were interested in accessing long-term diagnoses in their PEPA and information on their appointments. Accessing information on diagnostics and medical results including laboratory findings from different health care settings concerning the patients’ treatment were perceived as crucial by patients as well as both professional user groups. Information related to the patients’ medical history was mentioned several times. Patients perceived information regarding past family history like information on tumor diseases or congenital disease as relevant PEPA contents, whereas participants from the other HCP focus groups emphasized the importance of getting information on the patients’ social history. Participants from both the patient focus groups and the physician focus groups thematized the potential of information regarding the patient that could be made available through a PEPA. A section for information on internal professional documentation in a PEPA was seen as critical especially by the group of other HCPs. They highlighted the need for HCPs to be able to exchange information with each other on the patients’ condition or behavior without general access being available.

### User Requirements on Personal Electronic Health Record Functions

#### Overview

Identifying user requirements on PEPA functions revealed several topics that the focus group participants in the three user groups addressed. In addition to storage of and access to desired personal health information including clinical data, some identified central functions of a PEPA were selected and are presented in Table 5.

**Table 5:** User requirements on PEPA functions.

Subcategory	Aspects	User group^a^
Patients add information	Information on subjective well-being	a/b/c
	Patients could add commentaries	b/c
	Demand for separate section for patient entries	b
	Consequences on liability	b
Filter information	Filter large amount of data is crucial	b
	Filter for currentness	a/b/c
	Filter for diagnosis/topics	a/b/c
Electronic communication	Ambivalence towards messaging with patients	b/c
	Communication among HCPs	b
	Pressure by permanent availability	b
	Patients’ high expectations and limited resources in time	b/c

^a^Which user group was responsible for the aspect presented: a=patients; b=physicians; c=other HCPs (eg, nurses).

#### Patients Add Information

One central issue discussed in all three user groups was the opportunity for patients to add information to a PEPA in addition to the HCPs. The usefulness of information on subjective well-being that patients could add to a PEPA was emphasized. Some patients expressed the wish to share information on their well-being with their HCPs. Participants from both professional user groups (medical and other HCPs) gave examples on information they perceived as clinically relevant that they would want to know from the patient (eg, information on pain, depressive feelings, and side effects from the chemotherapy like nausea): “Well, yes, it’s data that the patient might generate themselves…It would be important to know that in terms of pain treatment, for instance” (GP2-F10).

A participant from a physician focus group suggested providing this function as a patient journal that would be kept by the patient in the PEPA: “Of course, that would be especially important, yes, if he could write a kind of journal…while he’s undergoing chemotherapy about how it’s going” (GP1-F09).

Furthermore, participants from both professional user groups mentioned the possibility for patients to add commentaries on medical results. For instance: “add something to it, a note/comment/commentary, if he wants, so that someone else who accesses it can either take it into account or not” (GP1-F05).

Critical comments were also made regarding patients’ adding information. From the physician group, requirements for a separate section for patient entries were identified to ensure that data entered by patients are not mixed with those uploaded from professionals and further to ensure that patient-added information would be marked as such:

Is that really necessary?...Then we’d need to enter certain codes for the patient to show where in his record… such as Patient History, where he can only enter something himself there and not add in other things.

GP1-F10

Given the fact that several physicians from different health care settings would have PEPA access, participants from physician focus groups expressed concerns and uncertainty regarding negative consequences on professionals’ liability for reacting to patient-added information or commentaries: “In theory you could go even further with the liability thing, if three or four doctors check the record and all saw that he was feeling worse and no one reacted, whose fault is it?” (GP1-F09).

#### Filter Information

Having the possibility to filter information in the PEPA was important to focus group participants from all three user groups: “But then there should be a filter function so you can filter out things fairly quickly, that would be important” (GP1-F09). Especially in terms of handling large amounts of data that a PEPA could provide, physicians perceived a filter function as essential: “The data volume soon won’t be a problem…but you need to be able to filter really well.” (GP4-F09).

Related to this topic, physicians perceived a filter function for currentness as useful, for example, listing medical results from the latest to the oldest: “Yeah, yeah, yeah. Well, I think the detailed filter function is very important too, eg to search for the latest results.” (GP2-F09).

Also, having the opportunity to filter the PEPA information for a diagnosis, for example, colorectal carcinoma or a topic of interest was highlighted as useful:

and so I can say, only colorectal carcinoma, then I only see everything connected with this diagnosis. If he comes to me because of high blood pressure, then I click at the top on just this episode and only see these things, that would be the benefit of it.

GP1-F10

#### Electronic Communication

Electronic communication could be a PEPA feature provided to its users. Among both HCP user groups, especially among physicians, ambivalence towards messaging with patients was revealed during the discussions. Some participants perceived this function as useful whereas others did not: “A kind of messaging function, being able to send messages to the patient would be useful: ‘Attention: please do not eat anything before attending your CT appointment tomorrow’” (Physician1-F04) and “That doesn’t make any sense” (GP5-F05).

Some physicians saw the positive potential of this feature for enhancing communication among HCPs involved in the patients’ treatment: “And perhaps sometimes a better coordination or a note to the GP as the most important second contact, leaving short messages via both channels without having to hold long phone calls and so on. This would certainly be beneficial” (Physician3-F04).

Concerns regarding electronic communication with patients that were expressed by physicians during the discussions referred to a perceived pressure by permanent availability of HCPs: “And if I then need to look up who just wrote to me and who I need to write to now because something urgent has happened, then I don’t need an emergency service any more, then I’m permanently on call” (GP1-F10).

It was argued that patients would develop high expectations concerning the HCPs’ availability in terms of dealing promptly with patient concerns, if such communication features would be available. They feared that their limited time resources could be overstrained by providing this feature to patients:

They can order prescriptions from us by e-mail and pick them up the next day. It’s actually a great function, but the problem is that patients send prescriptions at midnight and want to have them ready at 7 a.m. They think we sit here all day just for that.

HCP3-F07

## Discussion

### Principal Findings

The aim of this study was to explore requirements and needs of potential users with regard to the content and function of a PEPA. In our study, key requirements and needs were identified from users’ perspective. Overall, our participants were very much focused on themes according to their daily routines. They wished for a PEPA that allows and facilitates tracking the course of illness and treatment over time. Professionals expressed the need for presenting PEPA information in chronological order for their daily practice in treating patients with colorectal cancer. In this context, one central desired function to our participants was the opportunity to filter information and thus to categorize information for several purposes like time or content.

Physicians specifically highlighted the potential of a PEPA if it would enable them to view all information as history related to one single episode, for example, colorectal cancer. Closely linked with this requirement was the need to identify and utilize unstructured information and present it in structured form. Currently, a problem in daily practice described by professionals is that a lot of necessary health information is provided in an unstructured way, for example, in medical reports or physician’s letters (as PDF file). A real benefit for their daily work would be if a PEPA could identify and utilize unstructured information from various IT systems and present it in a structured form in a PEPA. However, there is a lack of literature addressing this issue [24].

A significant issue for patients and their HCPs was that PEPA information including medical information will be accessible to patients in their patient-controlled tool. Consequently, if patients managed their PEPA, they required that the presentation of data has to be adapted to the patient’s needs as a layperson, in particular, comprehensibility. Problems in understanding clinical documentation have often been an issue reported by patients [15,25-27]. Our patients wished to have a patient copy that is designed to be understood and handled by patients or wished a supportive glossary. As described previously in literature, patients want adapted patient versions of the record including reduced medical terms or support to quickly find definitions of medical terms [17].

In addition to storage and access to personal health information including clinical information [28,29], several desired PEPA functions were discussed. The idea that patients could autonomously add information to the PEPA was discussed by our participants. In general, patients were open in terms of this and found it was a good idea as supported by literature [12]. In particular, they were interested in adding information on their subjective well-being, for example, nausea during chemotherapy. However, adding general information about lifestyle choices such as exercise and smoking history was described as less interesting by participants from another study [10]. Most of our professional participants supported that this function could have additional benefit, for example, if it were in the form of a patient journal, in particular regarding clinical relevant information on patient well-being. However, some of them tended to be critical regarding the liability of professionals relying on patient-added information.

Despite the fact that electronic communication with HCPs is an often required and provided function [4,10,28-31] and higher satisfaction or improved doctor-patient communication was observed [32-35], our user groups did address this function, in particular patients. One possible explanation could be that patients in particular had no concrete ideas about electronic communication as a feature of patient-controlled records, due to the early stage of our PEPA development. Ambivalence towards messaging with patients was expressed especially by physicians. Their concerns referred to a perceived pressure by permanent availability. Moreover, this “electronic communication function" was seen more as a positive feature to enhance communication among HCPs involved in the patient treatment rather than a communication tool with patients. Experiences from a study with messaging services showed that patients most frequently rated the administrative communication functions as valuable features, such as the ability to request appointments, renew prescriptions, ask an administrative question, or obtain referral approvals. One third of respondents sent messages containing questions about their medical care [34]. Patient disappointment if professionals do not answer their questions has been described before [36].

In line with other studies, our findings referred to treatment-related information (eg, major diagnosis, information on allergies, medication lists, laboratory results, as well as to more general health information like social history, immunizations [2,5,8,10,37,38]). In particular, tumor-specific health information was important to patients with colorectal cancer and their HCPs. To oversee appointments was addressed by our patients as beneficial [28,29]. Information regarding the patients’ wishes were highlighted by both patients and HCPs. Similar findings are reported in a study of Patel [10].

Requirements regarding the layout were not specified by our participants, probably due to the early stage of technical development of the PEPA. However, our participants focused on general requirements like ergonomic issues, for example, clarity and ease of use. The ease of use of ICTs has been identified as a predictor for adoption [18,39-41], whereas complex portal interfaces were described as a barrier to use [8,42]. User-friendliness can be assumed as a central requirement for users’ adoption and use [2].

### Strengths and Limitations

As user acceptance has a significant impact on widespread implementation and use of a PEPA in health care, it is essential to involve users early in the technical development and evaluation processes in order to develop a patient/user-centered PEPA that addresses user needs. Consequently, exploring attitudes regarding the PEPA concept from the user perspective was an important first step in developing and implementing an innovative ICT into existing health care structures. The study was conducted by an interprofessional team of researchers (nursing, physiotherapy, medicine, philosophy) enabling a broad perspective during design and analysis stages. Some limitations in recruitment of participants have to be acknowledged. Most patients willing to participate in the study were men, and the level of education was relatively high. It can be assumed that the innovative and technical character of this approach attracted early adopters of ICT [4,8,43], therefore the findings may not be generalizable to the regional colorectal cancer patient population.

### Conclusions

From the user perspective, a PEPA was seen as a potentially useful tool for patients with colorectal cancer and their HCPs in cross-sectoral cancer care. A PEPA has potential to support patients in managing their chronic illness and is conceptualized to facilitate information exchange between patients and their HCPs as well as among HCPs or institutions across health care sectors. However, chronic diseases do have a long-term and episodic character. In order to create an added benefit to its users, a PEPA has to be oriented to these phases and episodes of care. A challenge for implementation will be how to design a PEPA’s health data in a patient accessible way. In order to create a real patient/user-centered tool that is tailored to user needs, patients and their HCPs have to be involved early in development, implementation, and evaluation processes. User preferences according to their daily routines in managing chronic illness have to be addressed. Furthermore, adequate patient support and technical advice for users have to be provided.

## Multimedia Appendix 1

Relevant information in a personal electronic health record from users' perspective linked with quotations and user requirements on relevant available information.

## Abbreviations

ECOG: Eastern Cooperative Oncology Group
GP: general practitioner
HCP: health care professional
ICT: information and communication technology
IQR: interquartile range
NCT: National Center for Tumor Diseases
INFOPAT: information technology for patient-centered health care
PEPA: name of the patient-controlled personal electronic health record which is developed within the INFOPAT project
PHR: personal electronic health record
