# Supplementary material for: Personal Electronic Health Records: Understanding User Requirements and Needs in Chronic Cancer Care
Source: J Med Internet Res. 2015 May 21;17(5):e121. doi: 10.2196/jmir.3884 (PMC4468571; doi:10.2196/jmir.3884)
Supplement: Supplementary file 1 [file jmir_v17i5e121_app1.pdf]

Multimedia Appendix 1

Legend of cited participants:

P=patient

PR= representatives from patient support groups

HCP= other non-medical health care professional, e.g. nurse

Ph= physician from hospital

GP= General practitioner

F01= focus group number 1; F02=focus group number 2; …

Quotations referring to box 1: Relevant PEPA information from users´ perspective

General	"That definitely needs to go in, allergies, diagnoses, medication and that's enough in principle. And basic data that is available at the interfaces." (GP2-F10)<br><br>"Well it needs to contain a core data set." (GP2-F10)<br><br>"Yes, we need to know about allergies, we want to know about current medication, erm, current state, I don't know, er, the basics." (Ph3-F10)<br><br>" […] What interests me most of course is the current diagnoses, the accompanying illnesses and the current medication, they're key points for me that I think would be quickest and most important when I look into the record." (Ph3-F10)
Long-term diagnosis	"Underlying illnesses such as diabetes, definitely." (P1-F02)<br><br>"Well, I'd want diagnostic data of course and the ability to pass it on to someone else, for example for a second opinion or something so that it doesn't need to be done all over again and I could say 'that's how things stand, what do you think?" (P4-F01)
Appointments	M: That means you want to have access to appointments, upcoming appointments.

	(Access to appointments, upcoming appointments) “Yes, that would be useful for me.” (P5-F03)
Medical results	“ […] Read about what they’ve done with me, operation reports or results from the CT with all the pictures. […] ” (P4-F01)<br>“I’m also interested in what they actually find out with the CT exams. If I get a CT done, I’d find out every quarter, let’s say, what is actually found out, it’ll be condensed onto a piece of paper.” (P4-F01)<br>“[…] I go for my routine GP check-up or I have something and he finds out this, that and the other. If I could then track what he did afterwards using this record– I don’t see any report from my GP.” (P2-F01)<br>“Well, results, radiology results, endoscopy results, histology, I suppose the software can label that fairly easily […] (Ph3-F04)
Laboratory findings	“Well I also get the lab results regularly from here (hospital), blood is taken by the GP. So I do get them, I’d want to have them in there (PEPA).” (P2-F01)<br>“As far as that’s concerned, I think lab results are a great thing. The GP takes the blood, we see it right away on our computers.” (HCP1-F06)
Family history	“And what I’ve been confronted with twice in connection with the cancer is that they asked me whether things had already occurred in the family. Well, even if they’re still alive and I can... they can’t remember because it goes back 2-3 generations. Such information could be important.” (P2-F01)
Social history	“A bit of background information concerning the social situation, how someone is looked after at home, whether there is family, whether he lives alone or whatever – these are often things that you find out very late […]” (HCP5-F06)
Medication	(Medication list) “Yes, that could be recorded, because like I said, I have it prescribed. You could do away with having to fill everything out every time.” (P6-F03)<br>“ […] the medication plan, everything up to date.” (GP4-F05)
Tumor specific information	“Date of the initial diagnosis, tumour stage, initial diagnosis.” (Ph1-F04)<br>“ […] Always the initial diagnosis of the tumour, then the brief histology and perhaps the localisation and mutation analysis and then what was done, always with the date. The most important examinations and the treatments given.” (Ph2-F04)

Information regarding the patient will	"Yes, health care proxy, that's a sub-area of the advance health care directive, who has the right to act on my behalf if I have dementia or something, that seems to be a more secure aspect to me. […] That's just something that occurred to me spontaneously, but the advance health care directives are important points." (P1-F02)<br><br>"Then you have a record that contains, I don't know, that the GP decides what to do in a palliative situation, that there is an advance health care directive, that he doesn't want any life-prolonging measures, no artificial feeding and so on, I don't know. It's a big help in the outpatient department." (GP1-F05)
Information on internal professional documentation	"Well to a certain degree, definitely not. […] You need to handle it carefully. There may be conversations that are written down so that the other carer knows about it and then the patient says 'I told them that in confidence and didn't want anyone else to know.' Such things need to be treated very carefully." (HCP3-F06)<br><br>"I think that this progression sheet should be left to us, but that if we write something specific, e.g. about a wound or something, then we can click a button and say: disclosed for patient record (PEPA), so that it appears in it […]" (HCP1-F06)

Box 2: User requirements on relevant available PEPA information

Categories	Contents	Specification
Personal information	Personal data	Name, date of birth, sex
	Information regarding the patient will	Advance health care directive
		Health care proxy
		Information about existence of advance health care directive or health care proxy
	Information on subjective well-being	Subjective well-being
		Pain assessment
		Assessment on depressiveness

	Information on appointments	-
Medical information	Allergies	-
	Status of vaccination	Electronic vaccination record
	Patients´ (past) medical history-	(past) Social history
		(past) Family history-
		Body size and weight
	Information on diagnostics	Physical examination
		Picture files of diagnostic measures
	Medical results	Laboratory findings
		Tumor marker, long-term findings on diabetes
		Radiological findings
		Medical report
		Reports from surgery
	Diagnosis	Current diagnosis
		Long-term diagnosis
		Diagnosis of pre-existing conditions
	Therapy	Current therapy
		Previous therapy
		Treatment plan

		Therapy recommendations
	Medication	Current medication<br>Previous medication<br>Current medication plan<br>Context information<br>(who prescribed, date of prescription)
	Information on internal professional documentation	Separate area for internal professionals` documentation
	Information on nursing documentation	Wound assessment and care
	Information on informed consent discussion	-
Extra Information	Information on all treating and caring persons	Name<br>Contact number
